# Supplementary figures and images for: Different Ras isoforms regulate synaptic plasticity in opposite directions
Source: EMBO J. 2025 Feb 21;44(7):2106–33. doi: 10.1038/s44318-025-00390-8 (PMC11961722; doi:10.1038/s44318-025-00390-8)

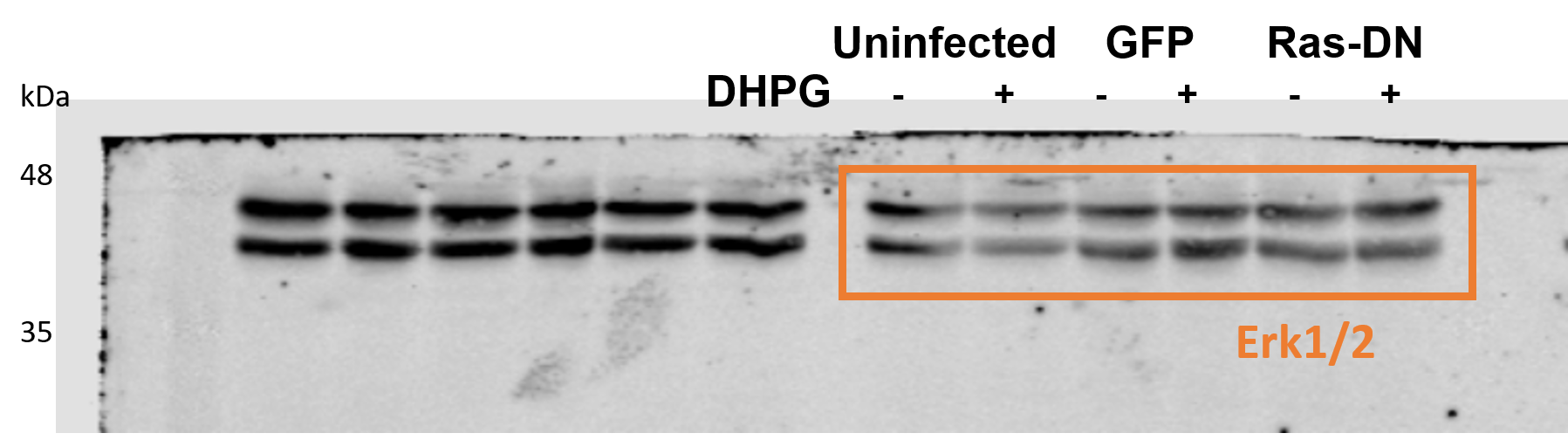

Supplement: Supplementary file 2 — Source data Fig. 1 [file 44318_2025_390_MOESM2_ESM.zip › Fig 1/1B/WB Erk.tif]

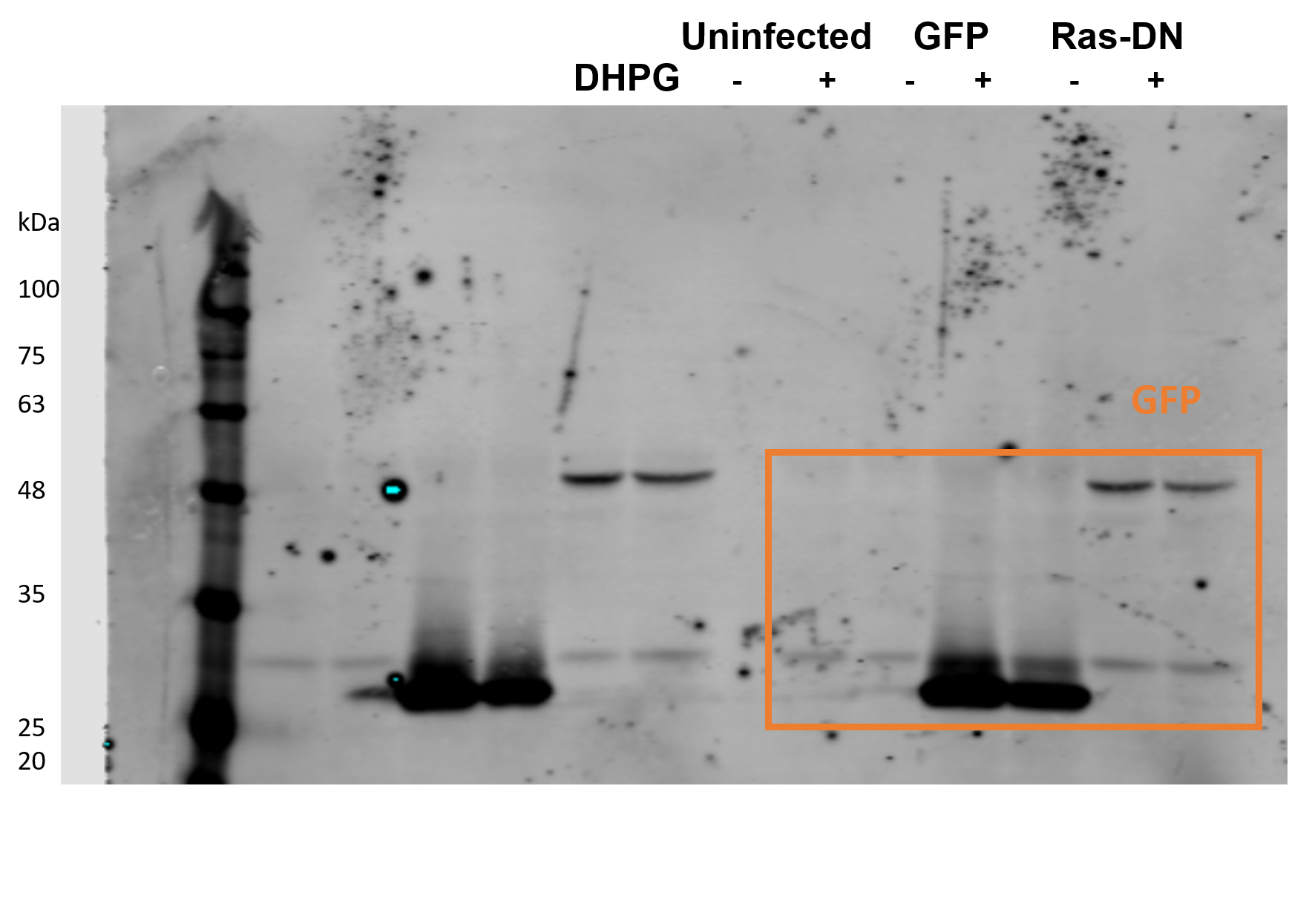

Supplement: Supplementary file 2 — Source data Fig. 1 [file 44318_2025_390_MOESM2_ESM.zip › Fig 1/1B/WB GFP.tif]

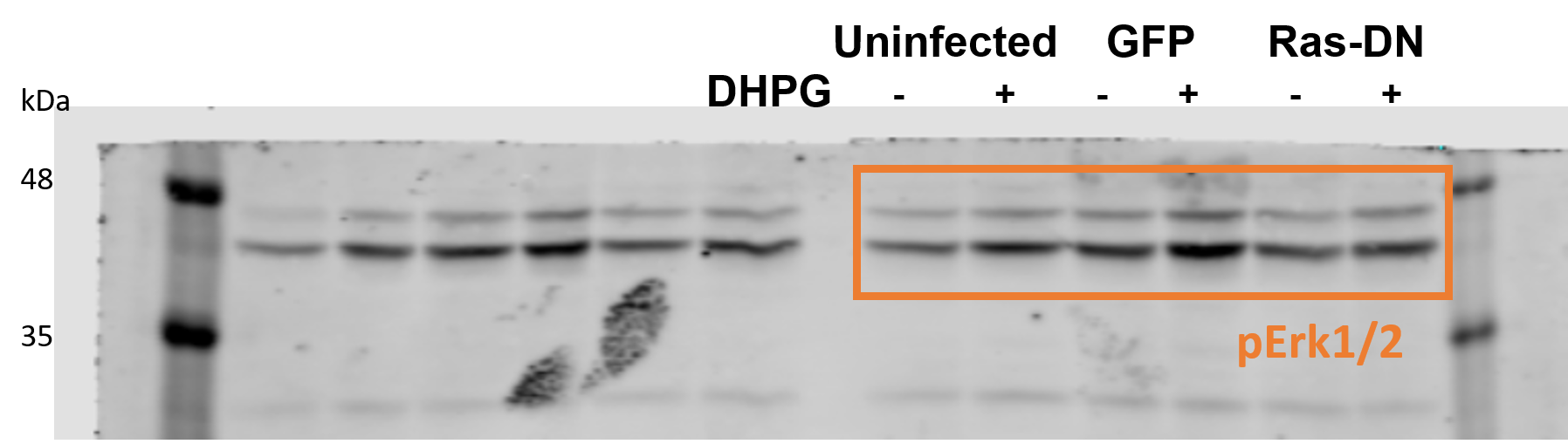

Supplement: Supplementary file 2 — Source data Fig. 1 [file 44318_2025_390_MOESM2_ESM.zip › Fig 1/1B/WB pErk.tif]

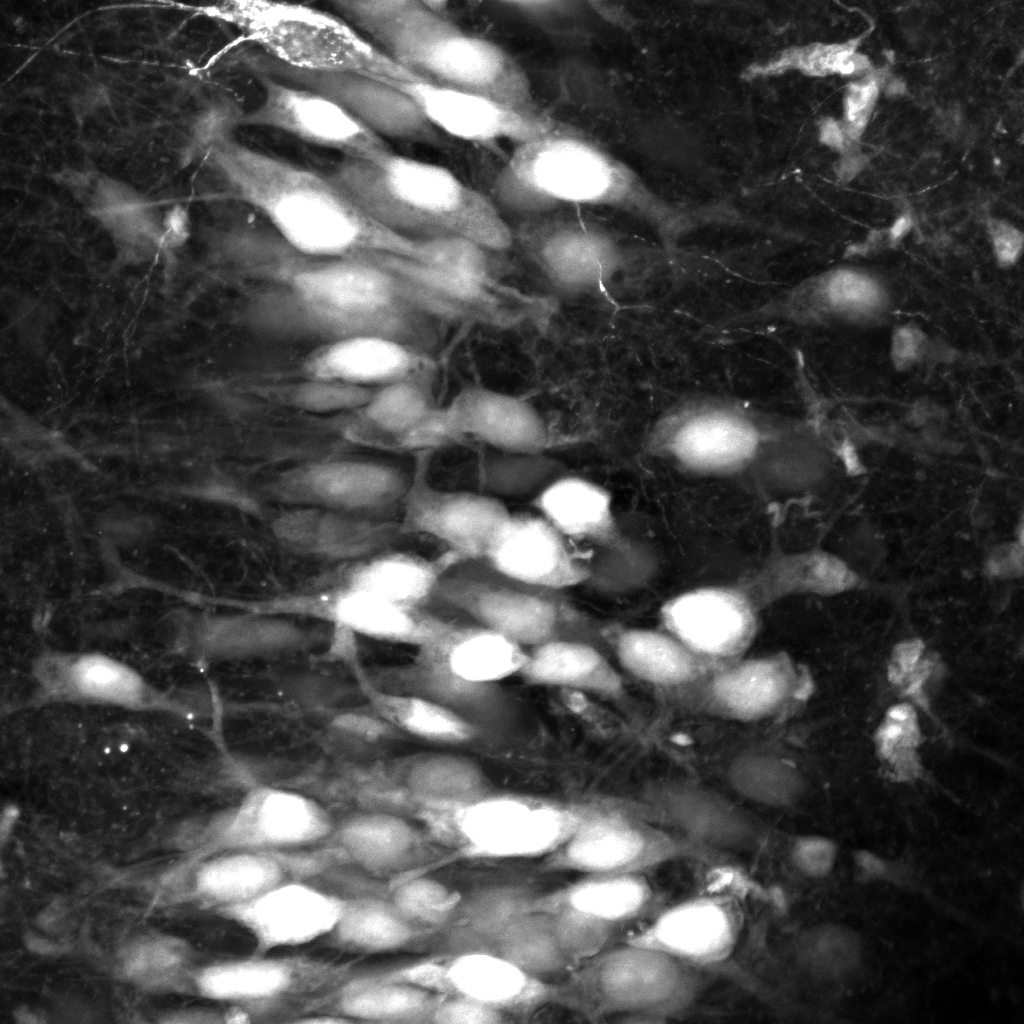

Supplement: Supplementary file 2 — Source data Fig. 1 [file 44318_2025_390_MOESM2_ESM.zip › Fig 1/1C/MAX_Control-merged.tif]

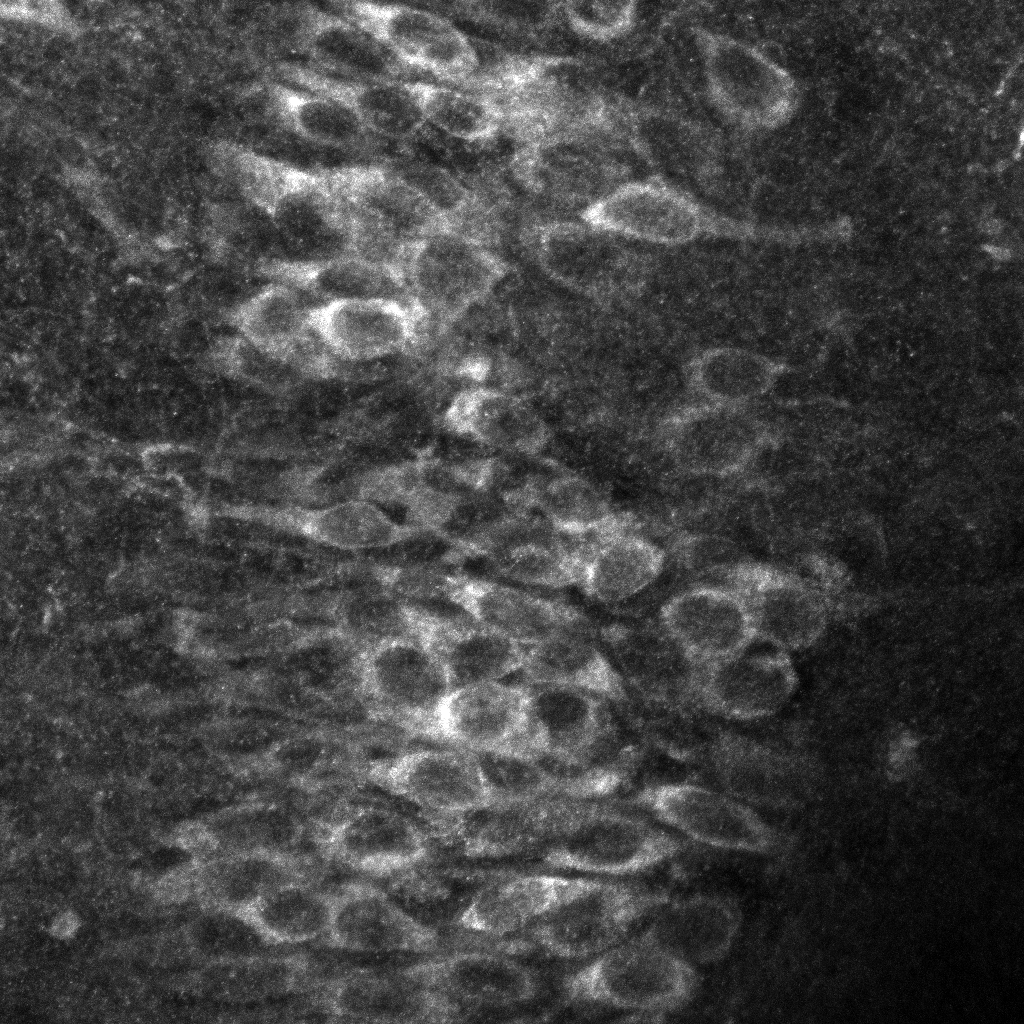

Supplement: Supplementary file 2 — Source data Fig. 1 [file 44318_2025_390_MOESM2_ESM.zip › Fig 1/1C/MAX_Control-puromycin.tif]

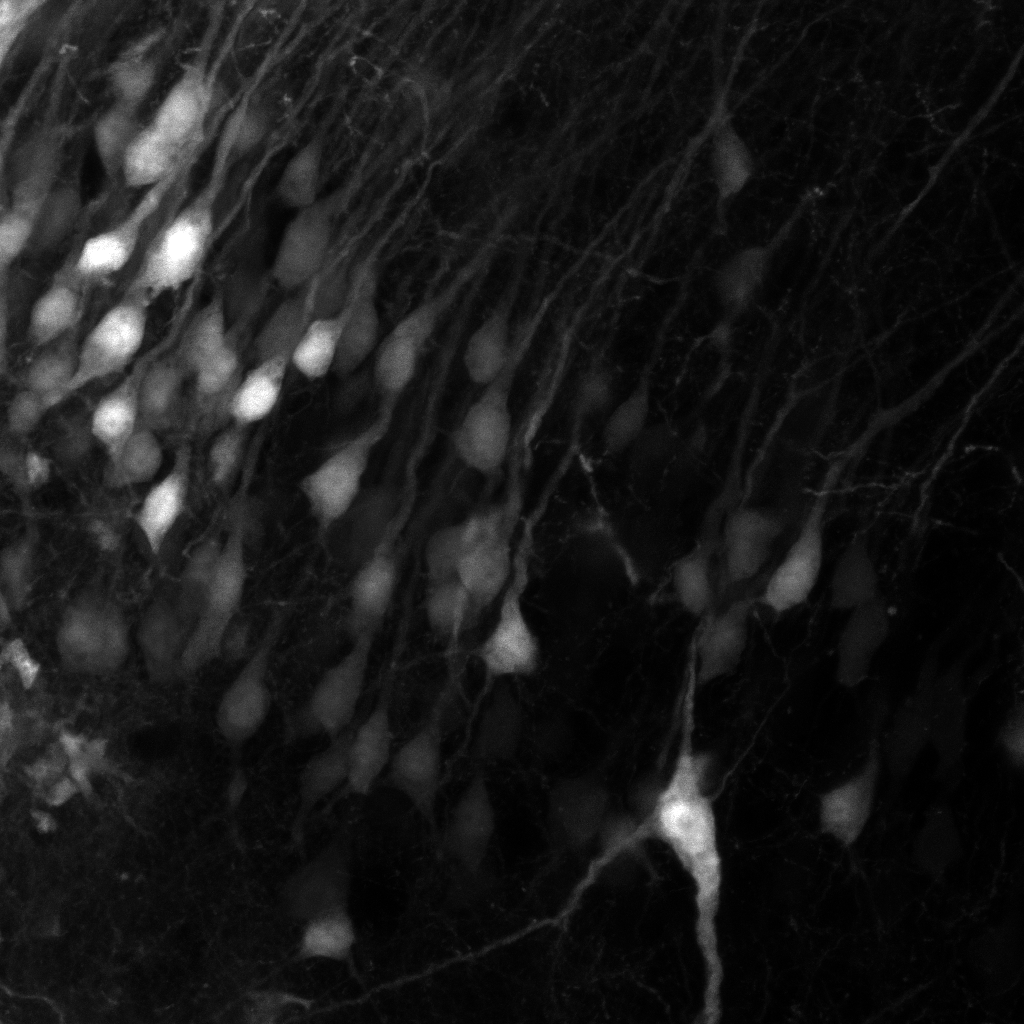

Supplement: Supplementary file 2 — Source data Fig. 1 [file 44318_2025_390_MOESM2_ESM.zip › Fig 1/1C/MAX_DHPG-merged.tif]

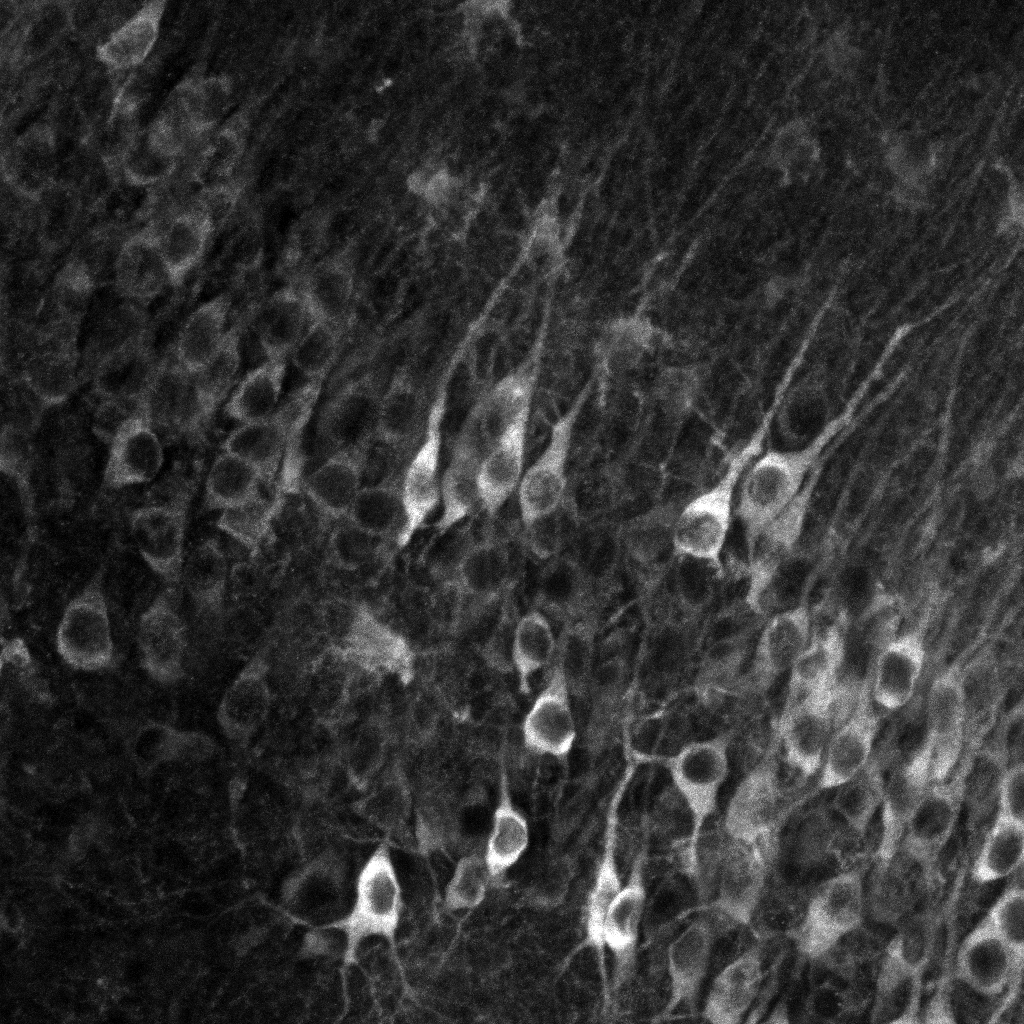

Supplement: Supplementary file 2 — Source data Fig. 1 [file 44318_2025_390_MOESM2_ESM.zip › Fig 1/1C/MAX_DHPG-puromycin.tif]

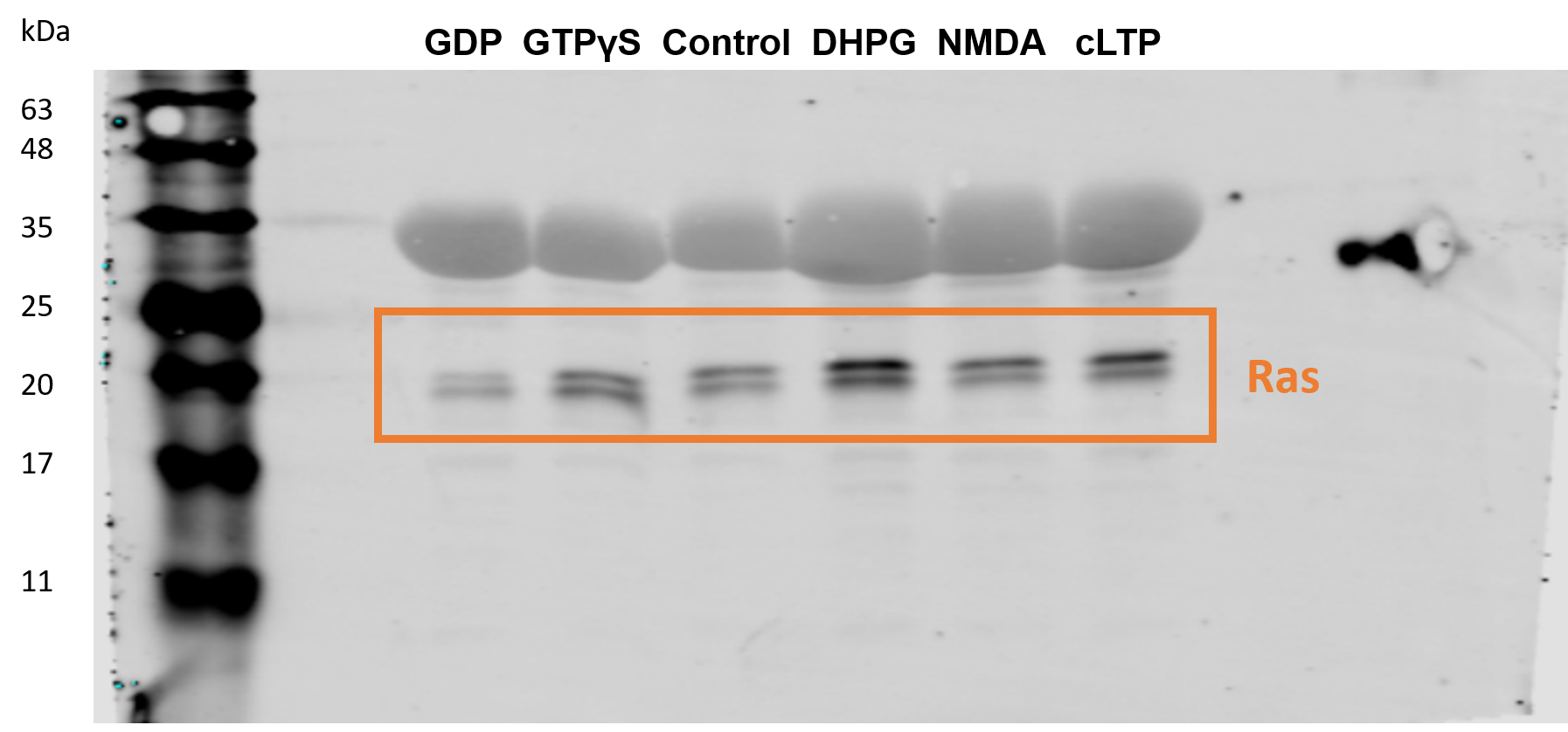

Supplement: Supplementary file 3 — Source data Fig. 2 [file 44318_2025_390_MOESM3_ESM.zip › Fig 2/2A/WB Ras Bound.tif]

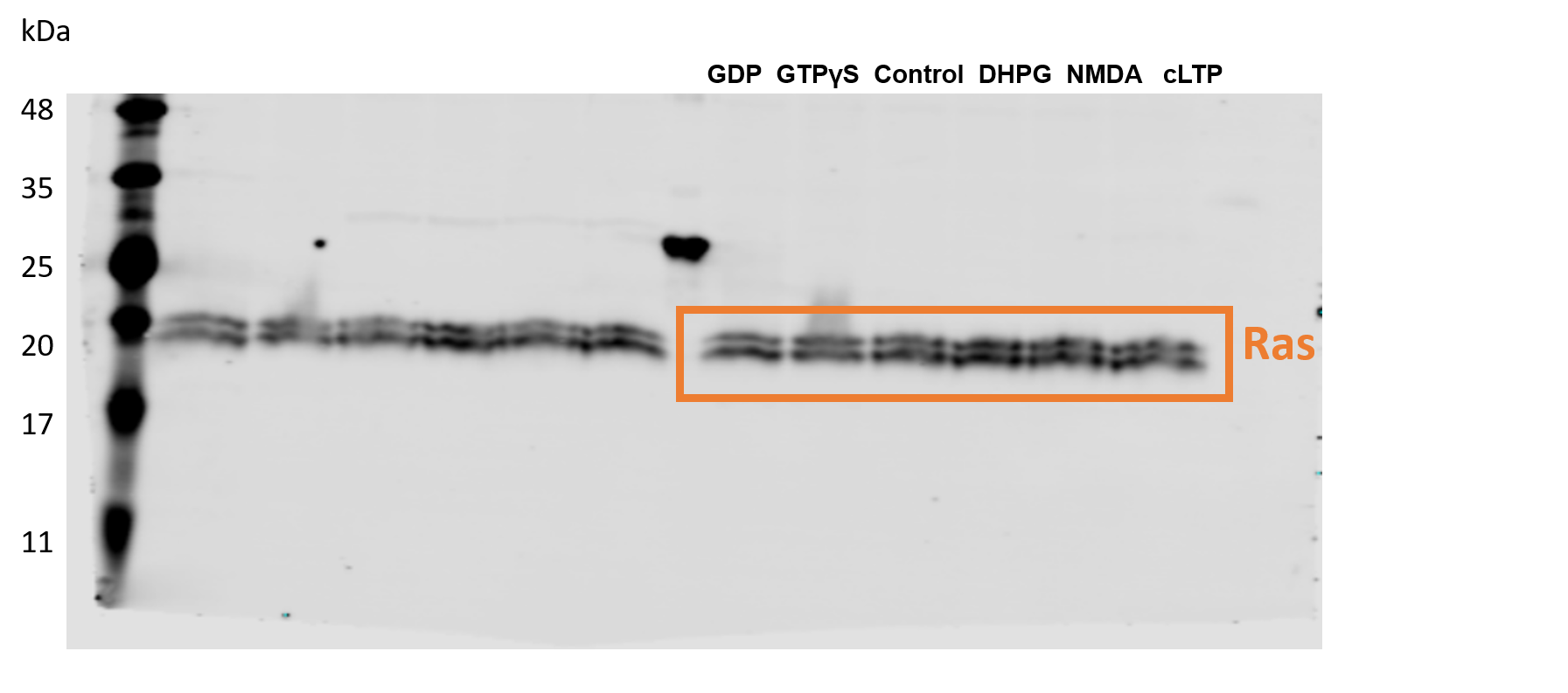

Supplement: Supplementary file 3 — Source data Fig. 2 [file 44318_2025_390_MOESM3_ESM.zip › Fig 2/2A/WB Ras input.tif]

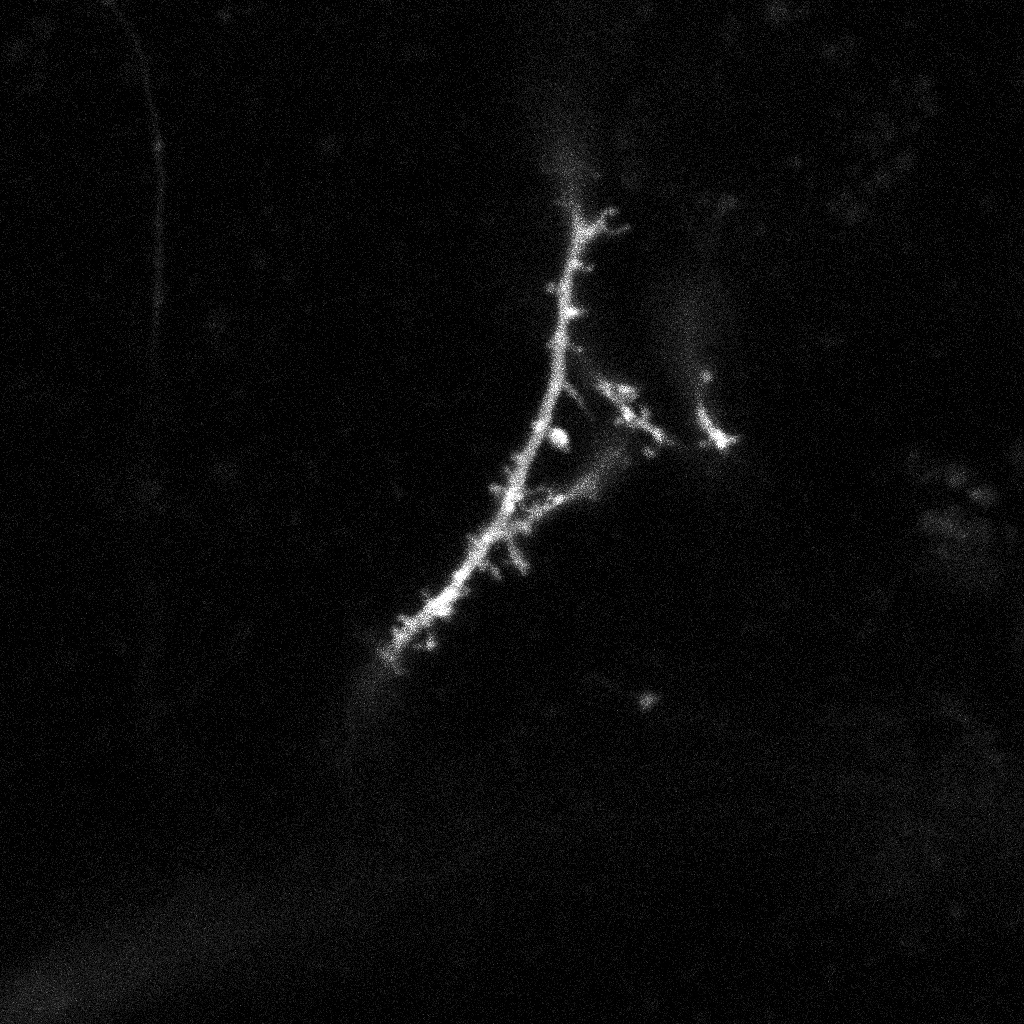

Supplement: Supplementary file 3 — Source data Fig. 2 [file 44318_2025_390_MOESM3_ESM.zip › Fig 2/2B/MAX_FRET_Baseline.tif]

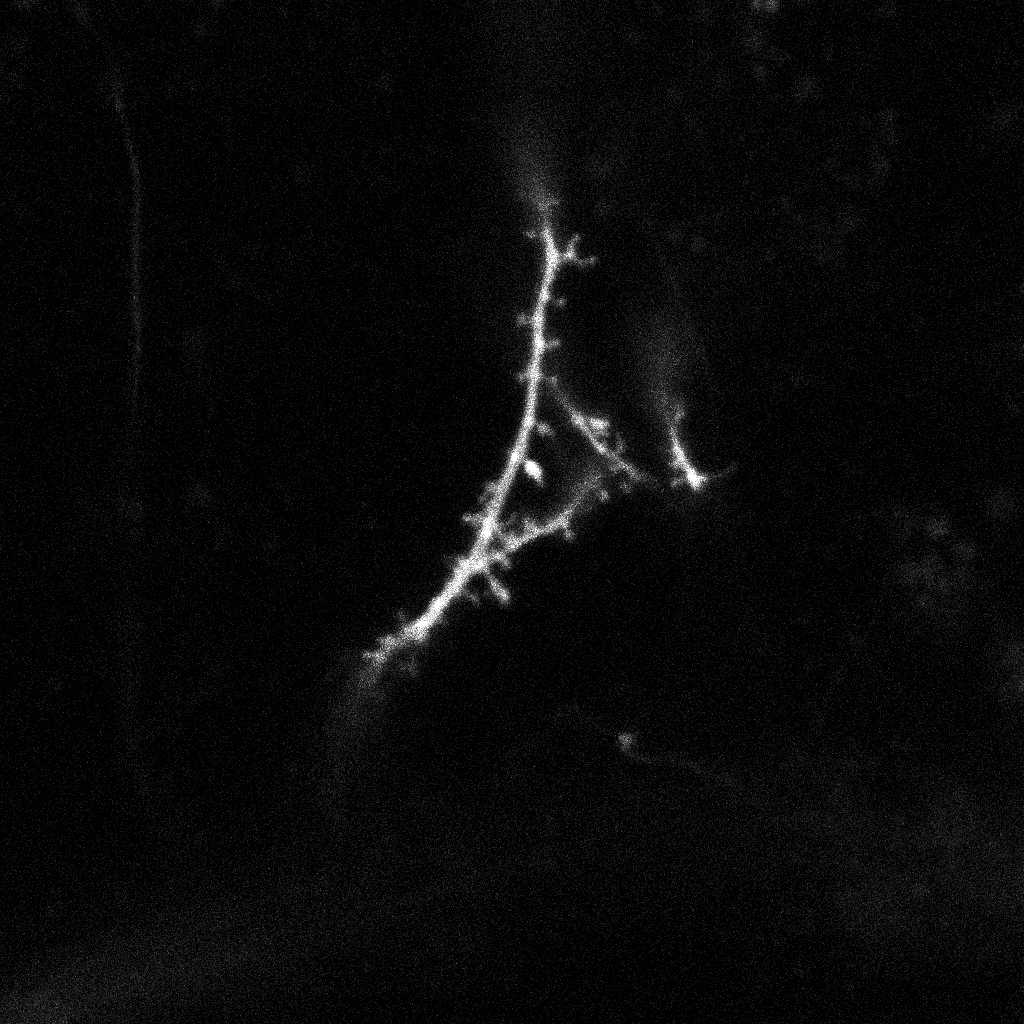

Supplement: Supplementary file 3 — Source data Fig. 2 [file 44318_2025_390_MOESM3_ESM.zip › Fig 2/2B/MAX_FRET_DHPG.tif]

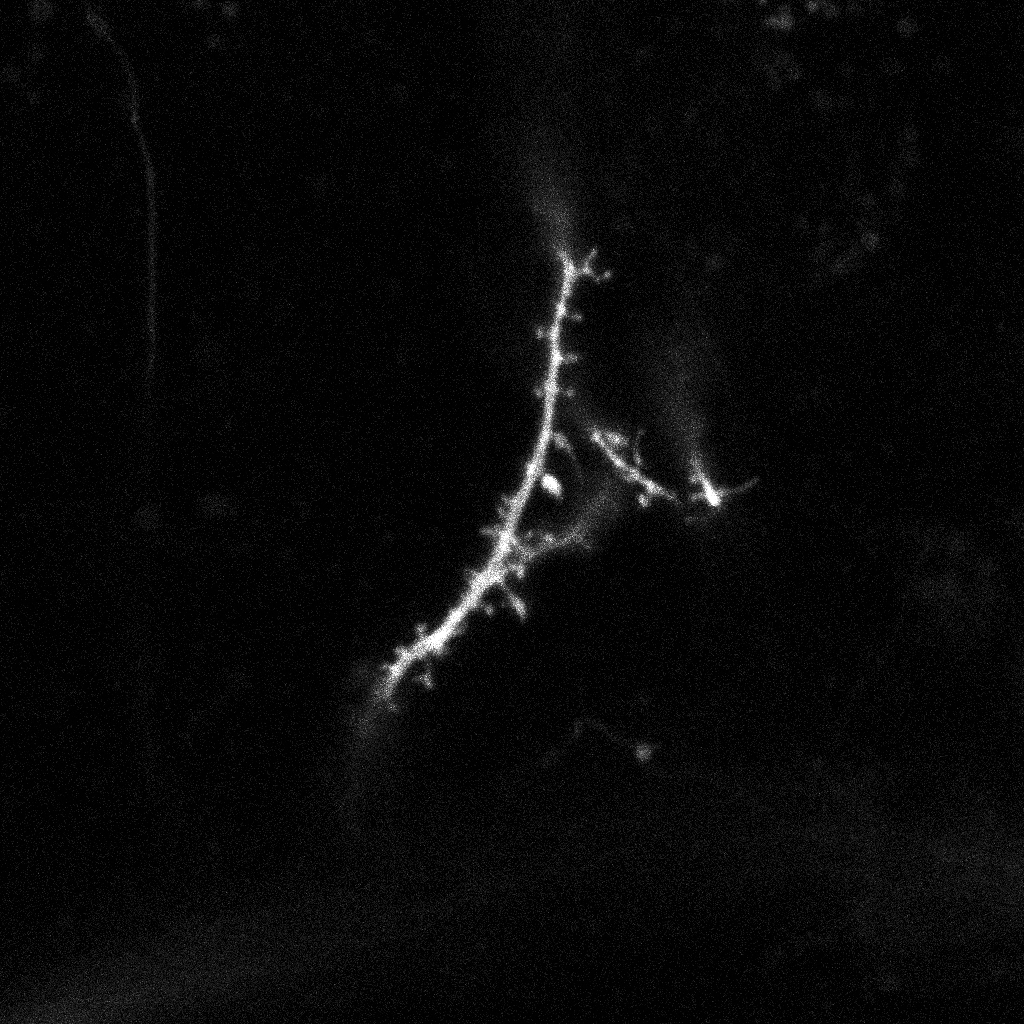

Supplement: Supplementary file 3 — Source data Fig. 2 [file 44318_2025_390_MOESM3_ESM.zip › Fig 2/2B/MAX_FRET_Wash-out.tif]

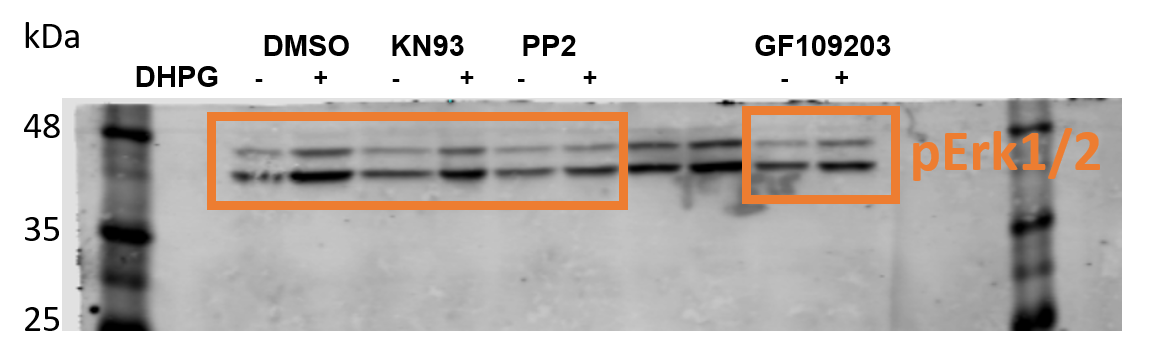

Supplement: Supplementary file 3 — Source data Fig. 2 [file 44318_2025_390_MOESM3_ESM.zip › Fig 2/2C/WB Erk.tif]

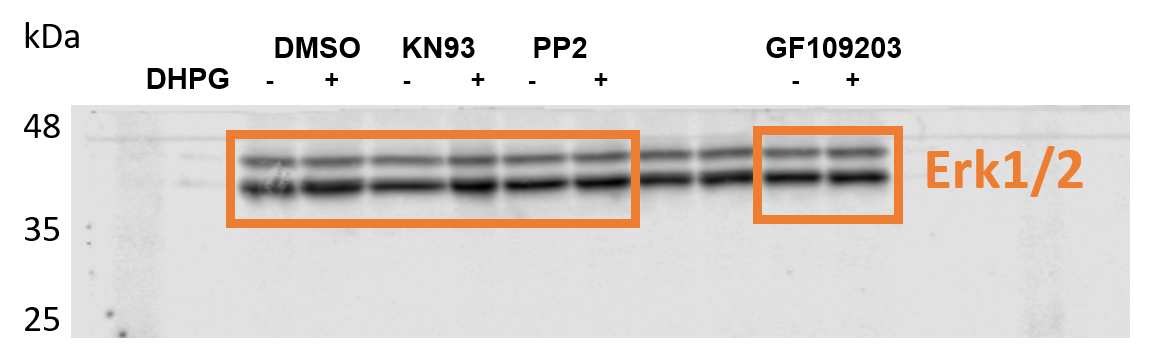

Supplement: Supplementary file 3 — Source data Fig. 2 [file 44318_2025_390_MOESM3_ESM.zip › Fig 2/2C/WB pErk.tif]

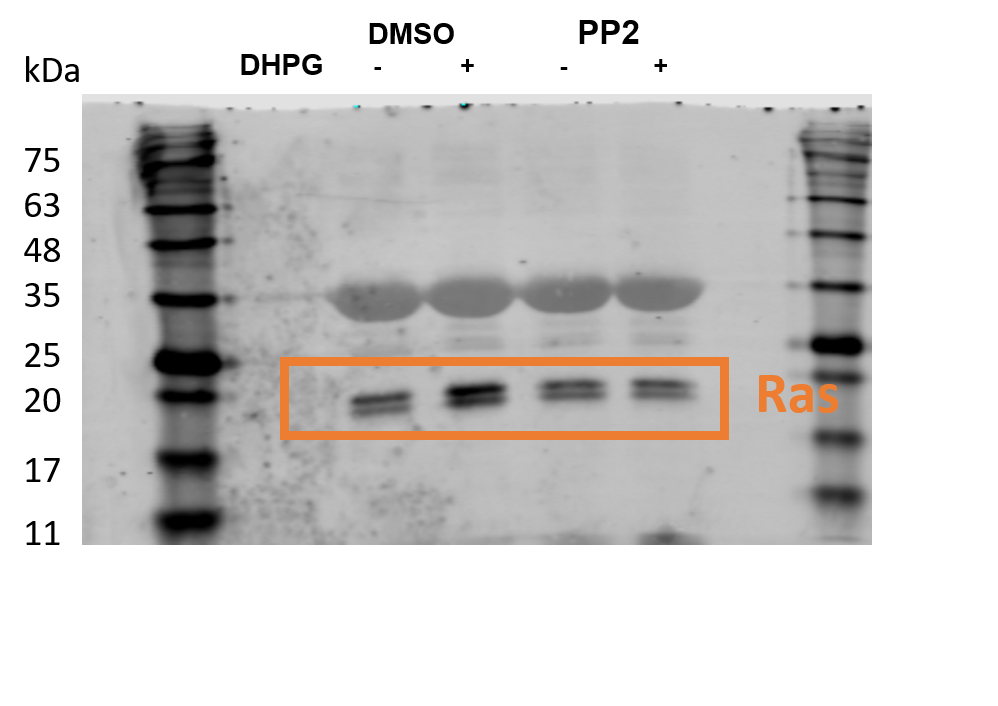

Supplement: Supplementary file 3 — Source data Fig. 2 [file 44318_2025_390_MOESM3_ESM.zip › Fig 2/2D/WB Ras Bound.tif]

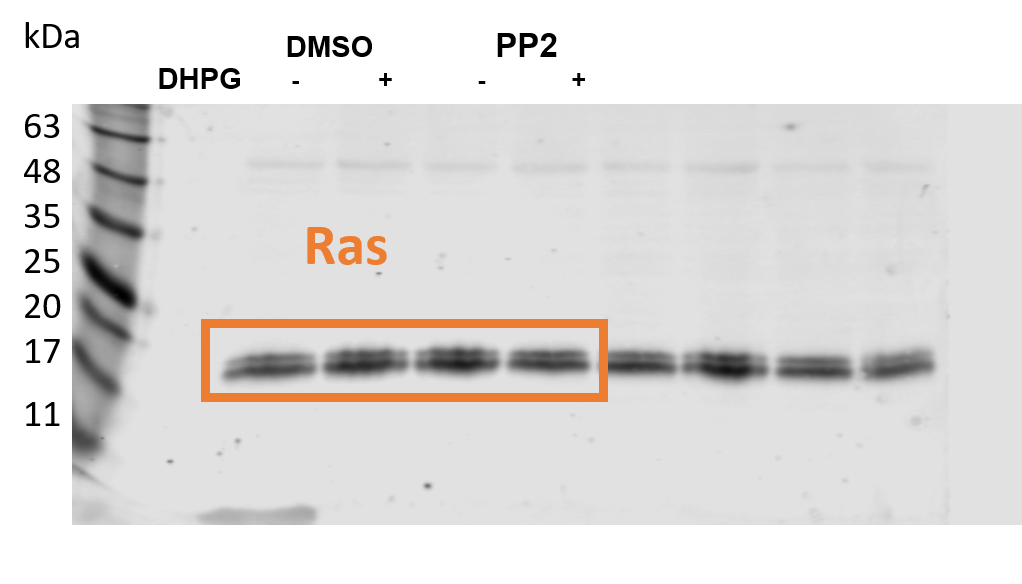

Supplement: Supplementary file 3 — Source data Fig. 2 [file 44318_2025_390_MOESM3_ESM.zip › Fig 2/2D/WB Ras input.tif]

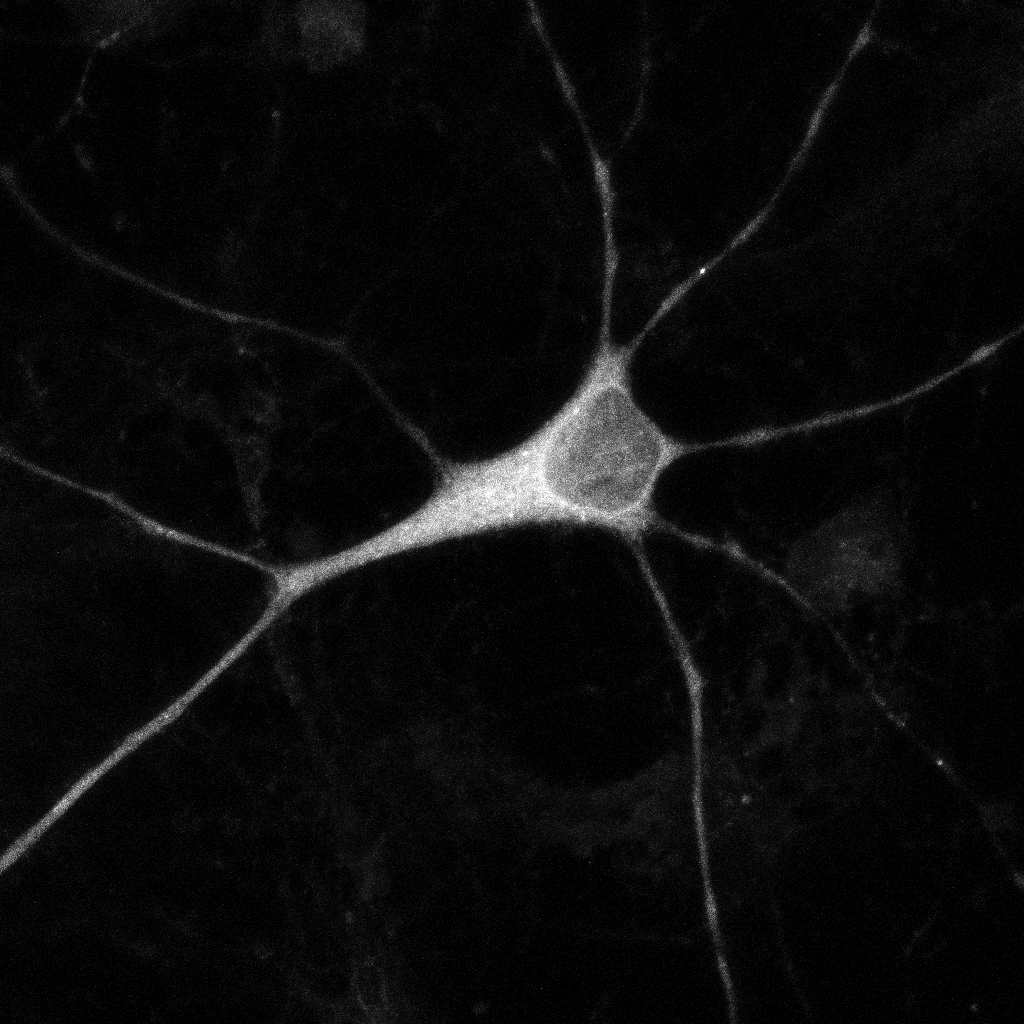

Supplement: Supplementary file 5 — Source data Fig. 4 [file 44318_2025_390_MOESM5_ESM.zip › Fig 4/4E/MAX_C181,184S_soma.tif]

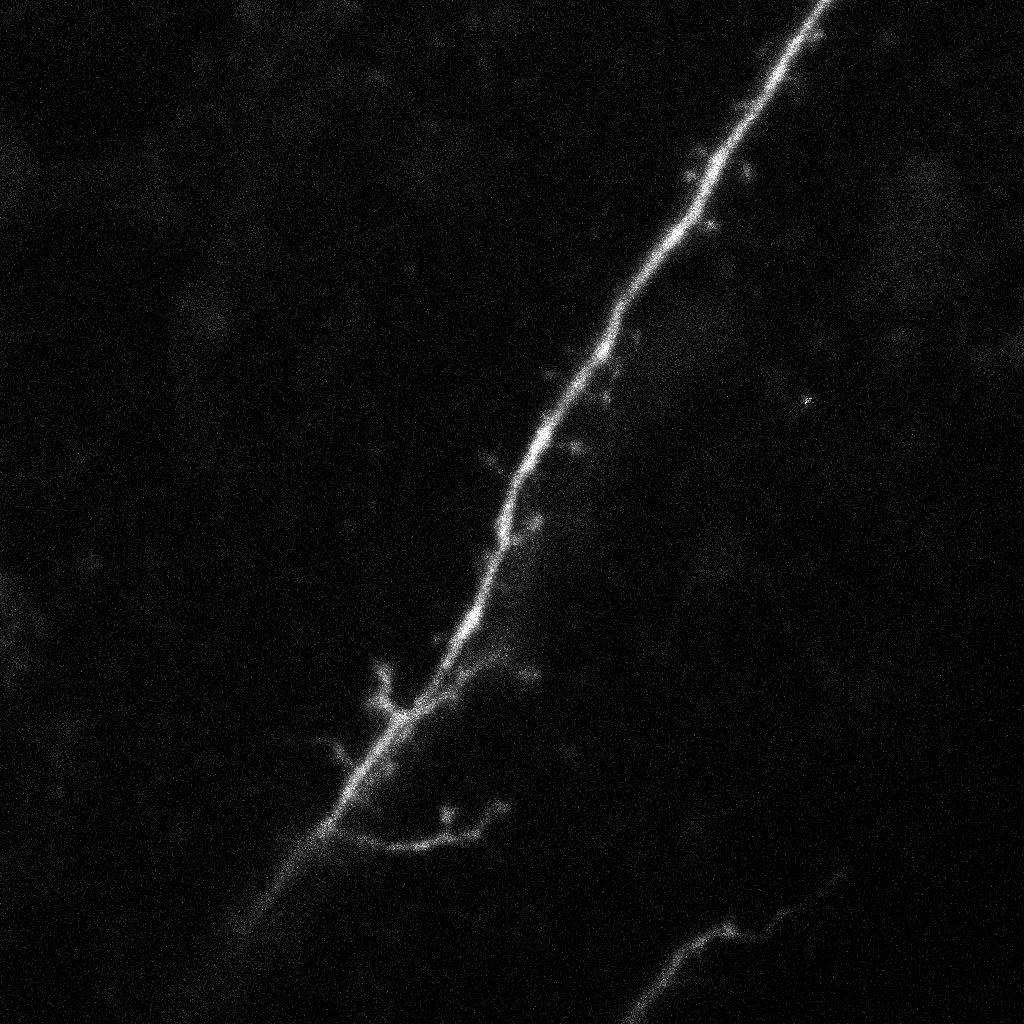

Supplement: Supplementary file 5 — Source data Fig. 4 [file 44318_2025_390_MOESM5_ESM.zip › Fig 4/4E/MAX_C181,184S_spines.tif]

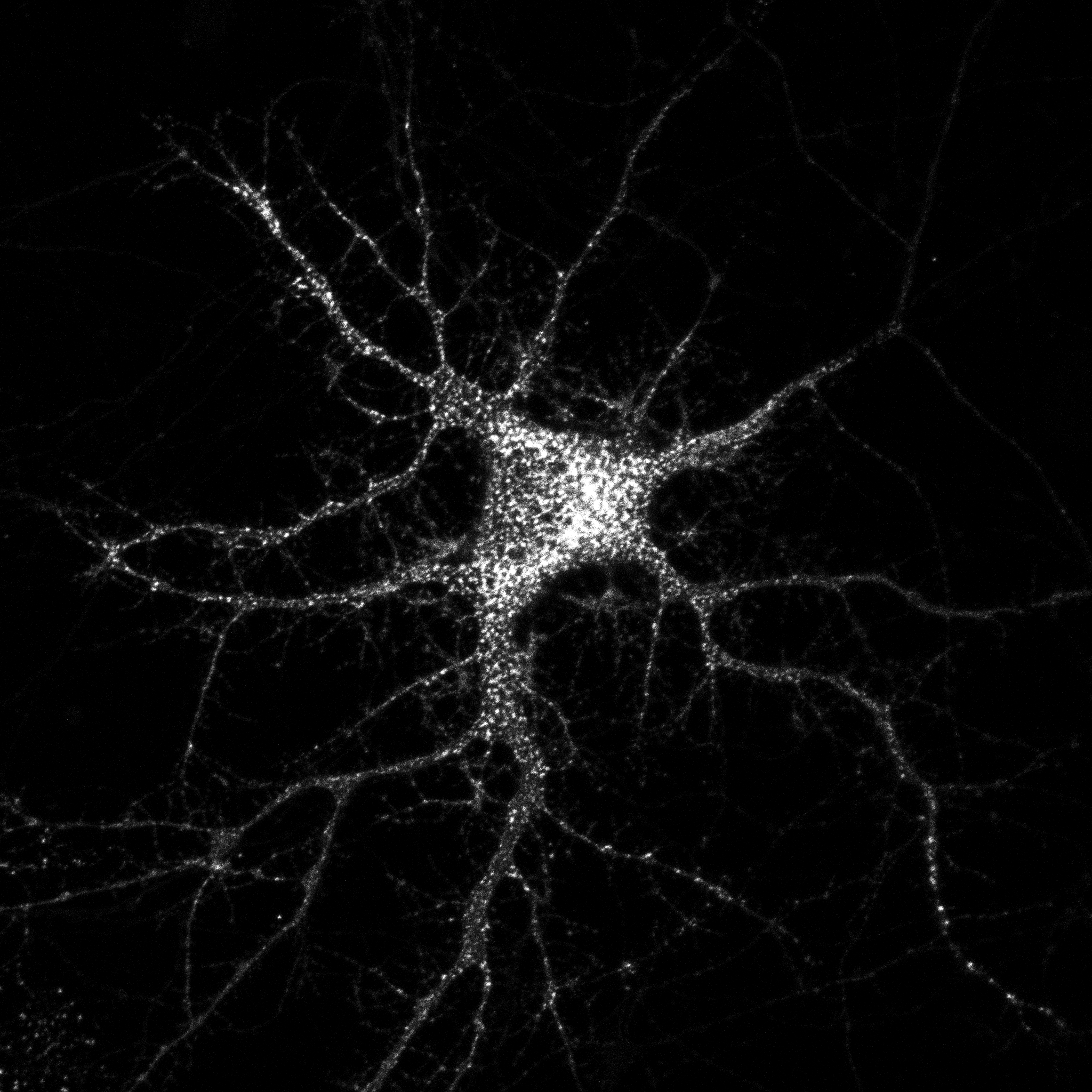

Supplement: Supplementary file 5 — Source data Fig. 4 [file 44318_2025_390_MOESM5_ESM.zip › Fig 4/4E/MAX_WT_soma.tif]

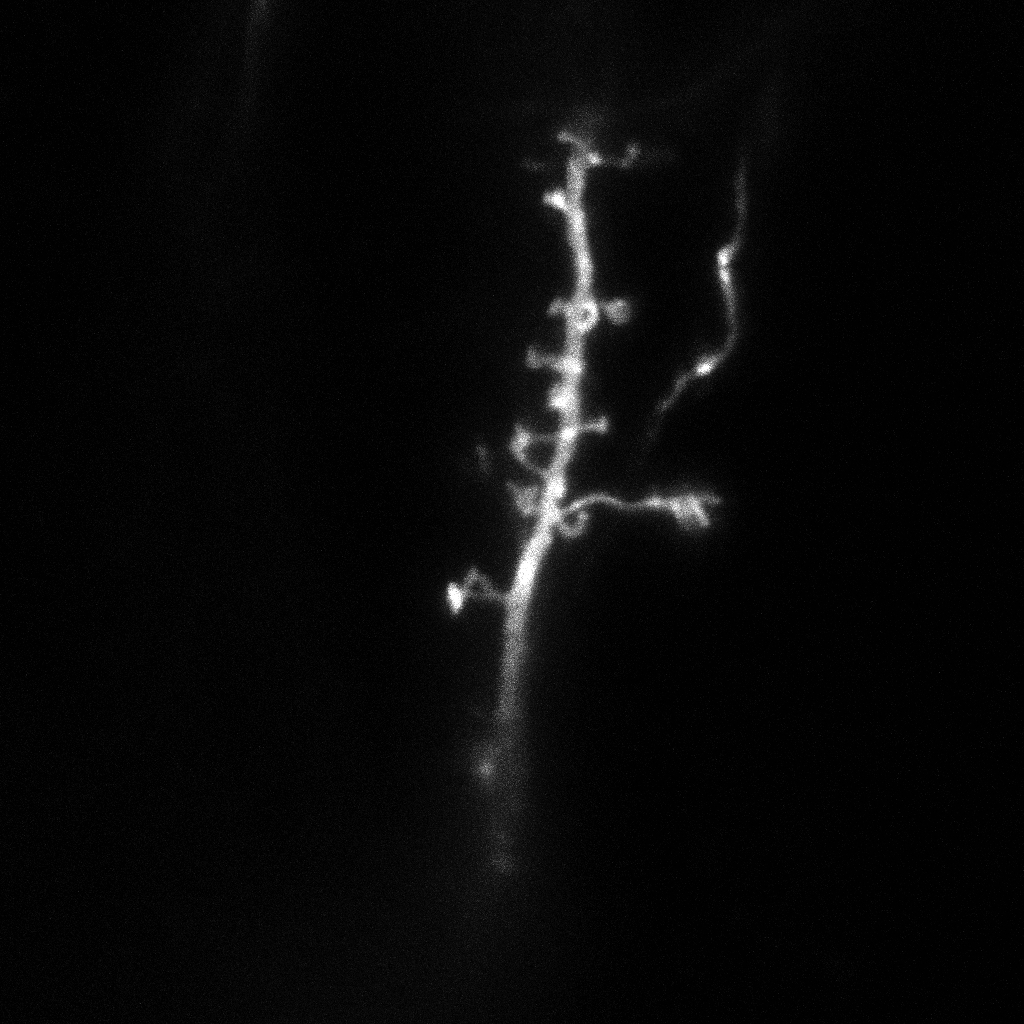

Supplement: Supplementary file 5 — Source data Fig. 4 [file 44318_2025_390_MOESM5_ESM.zip › Fig 4/4E/MAX_WT_spines.tif]

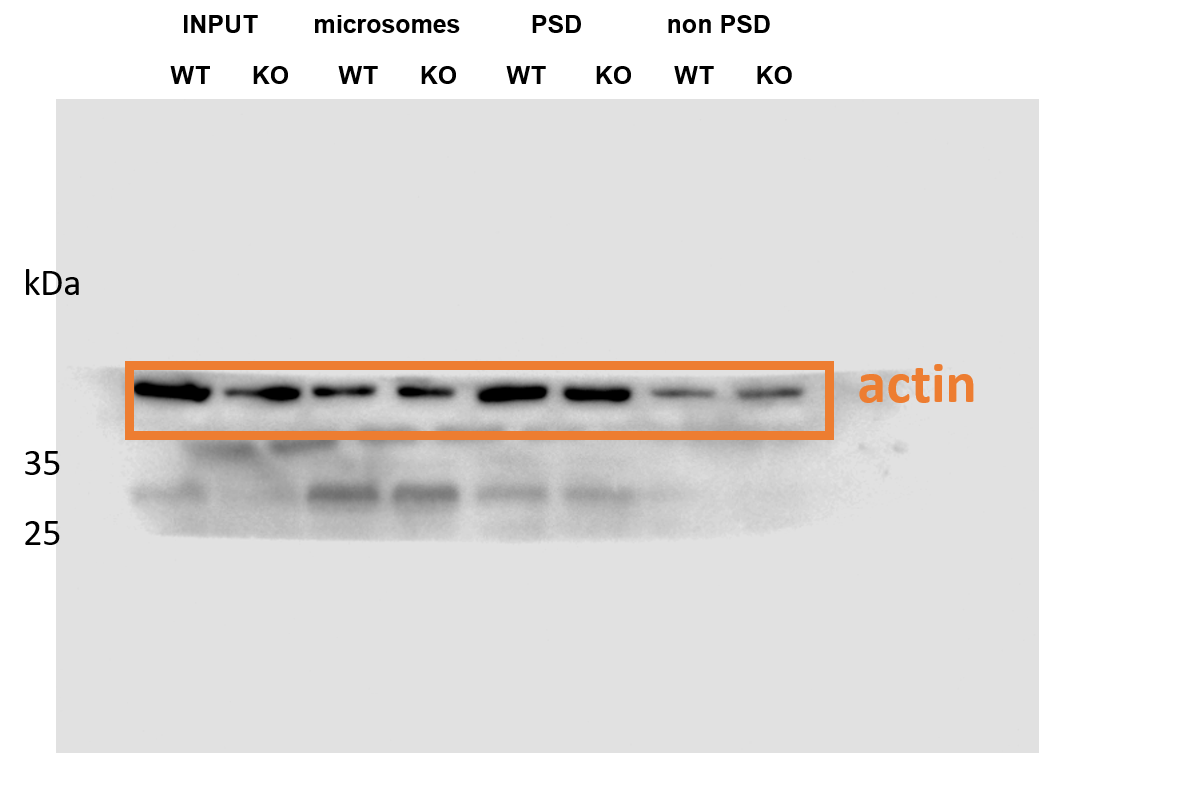

Supplement: Supplementary file 6 — Source data Fig. 5 [file 44318_2025_390_MOESM6_ESM.zip › Fig 5/5A/WB actin.tif]

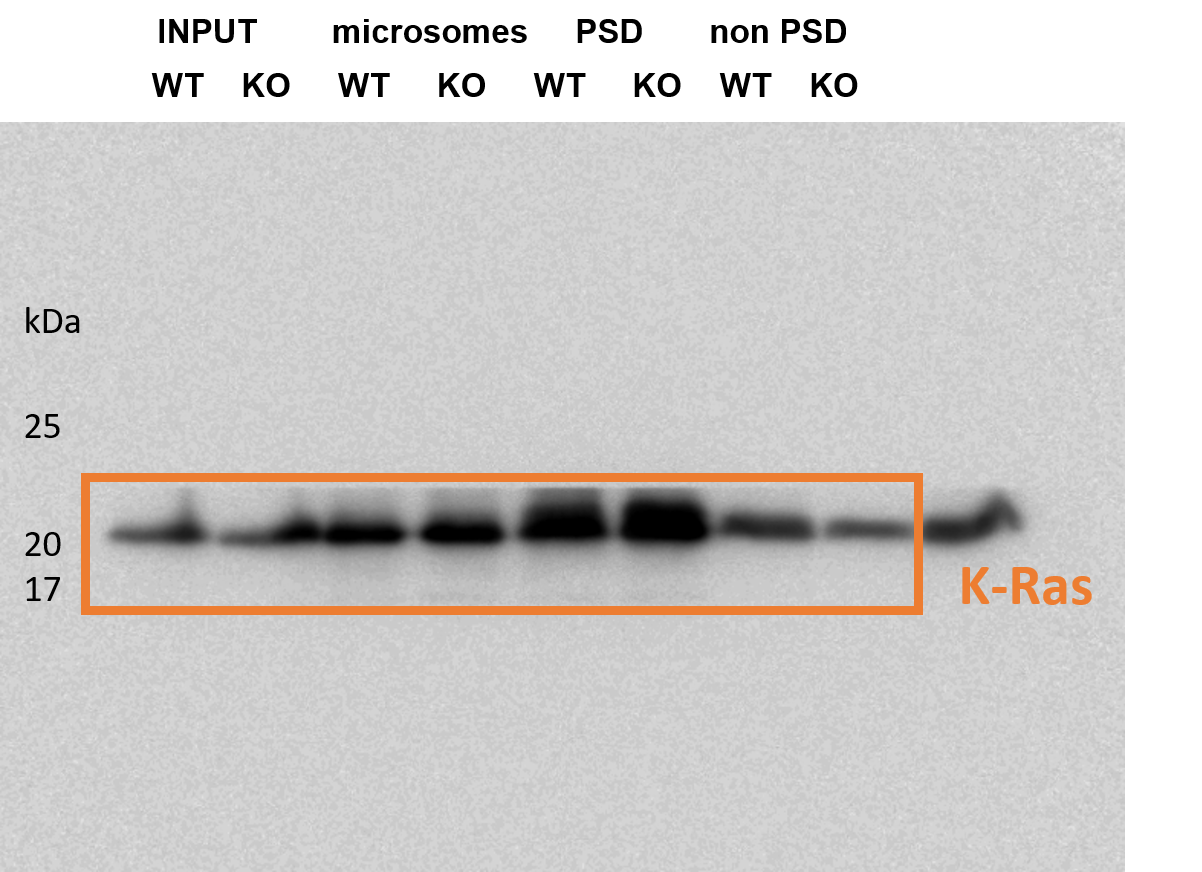

Supplement: Supplementary file 6 — Source data Fig. 5 [file 44318_2025_390_MOESM6_ESM.zip › Fig 5/5A/WB K-Ras.tif]

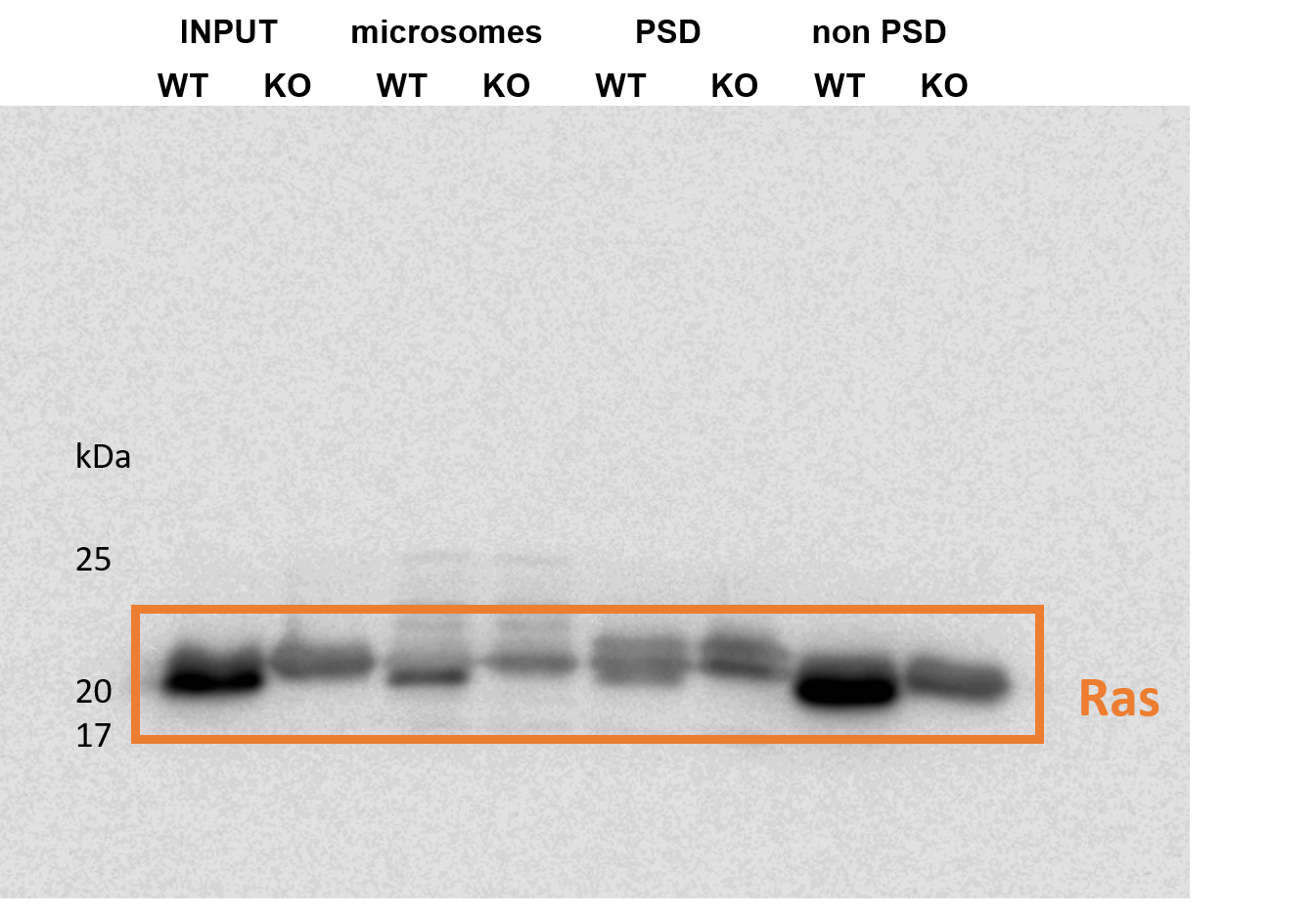

Supplement: Supplementary file 6 — Source data Fig. 5 [file 44318_2025_390_MOESM6_ESM.zip › Fig 5/5A/WB panRas.tif]

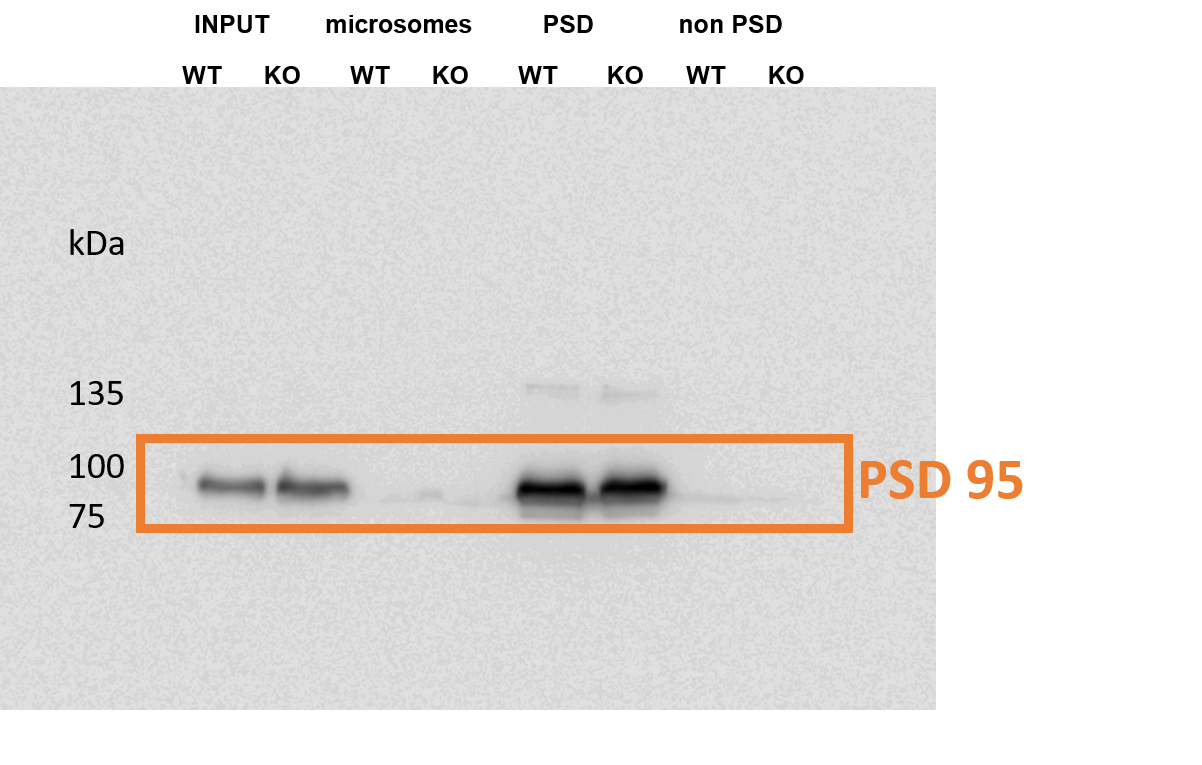

Supplement: Supplementary file 6 — Source data Fig. 5 [file 44318_2025_390_MOESM6_ESM.zip › Fig 5/5A/WB PSD95.tif]

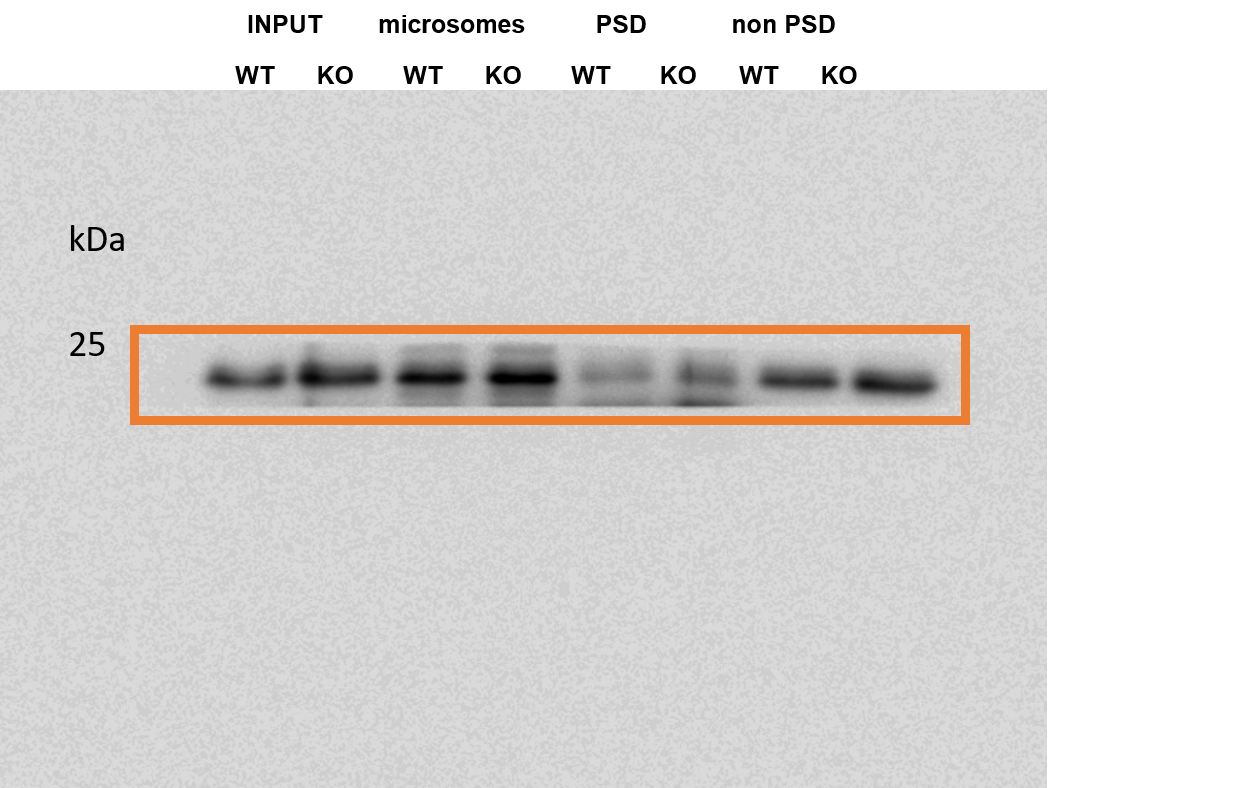

Supplement: Supplementary file 6 — Source data Fig. 5 [file 44318_2025_390_MOESM6_ESM.zip › Fig 5/5A/WB Rab5.tif]

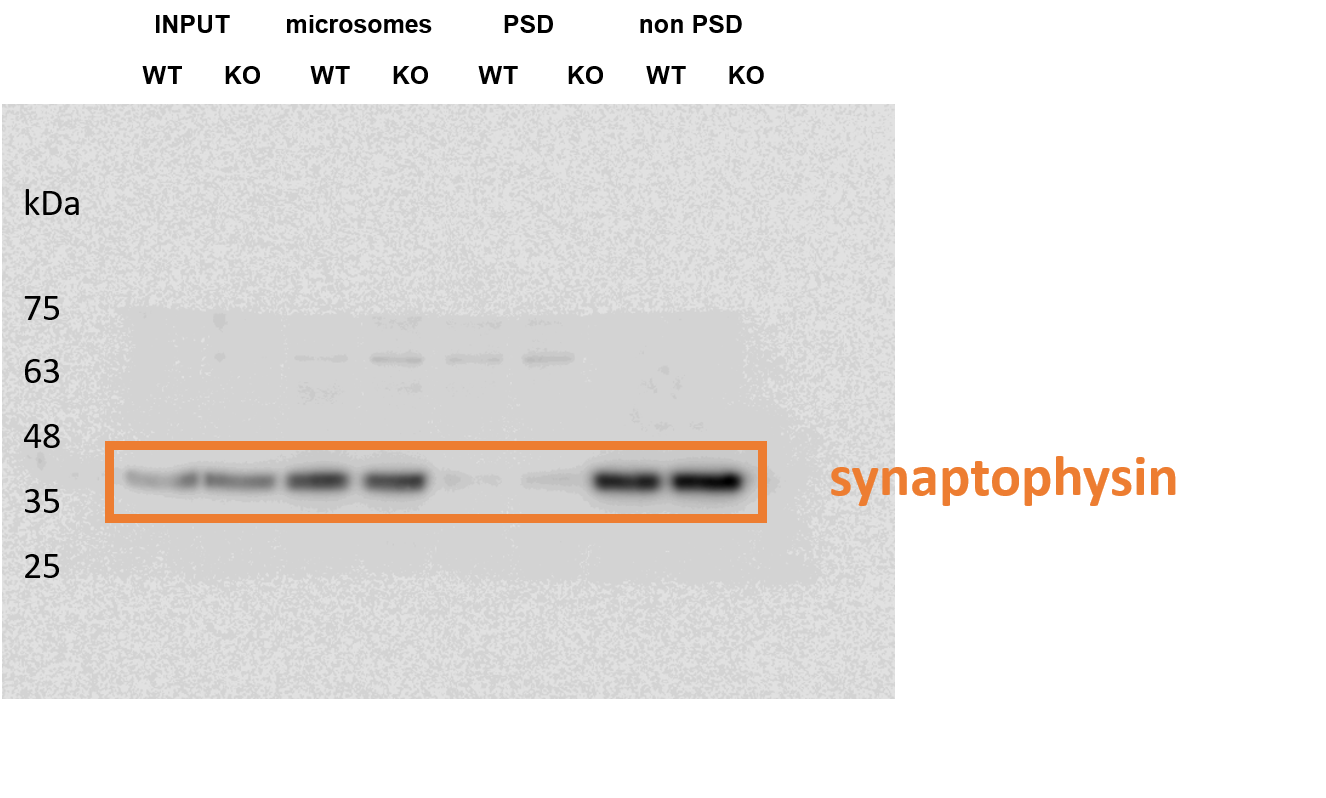

Supplement: Supplementary file 6 — Source data Fig. 5 [file 44318_2025_390_MOESM6_ESM.zip › Fig 5/5A/WB synaptophysin.tif]
