# Supplementary material for: Different Ras isoforms regulate synaptic plasticity in opposite directions
Source: EMBO J. 2025 Feb 21;44(7):2106–33. doi: 10.1038/s44318-025-00390-8 (PMC11961722; doi:10.1038/s44318-025-00390-8)
Supplement: Supplementary file 7 — Expanded View Figures [file 44318_2025_390_MOESM7_ESM.pdf]

Expanded View Figures

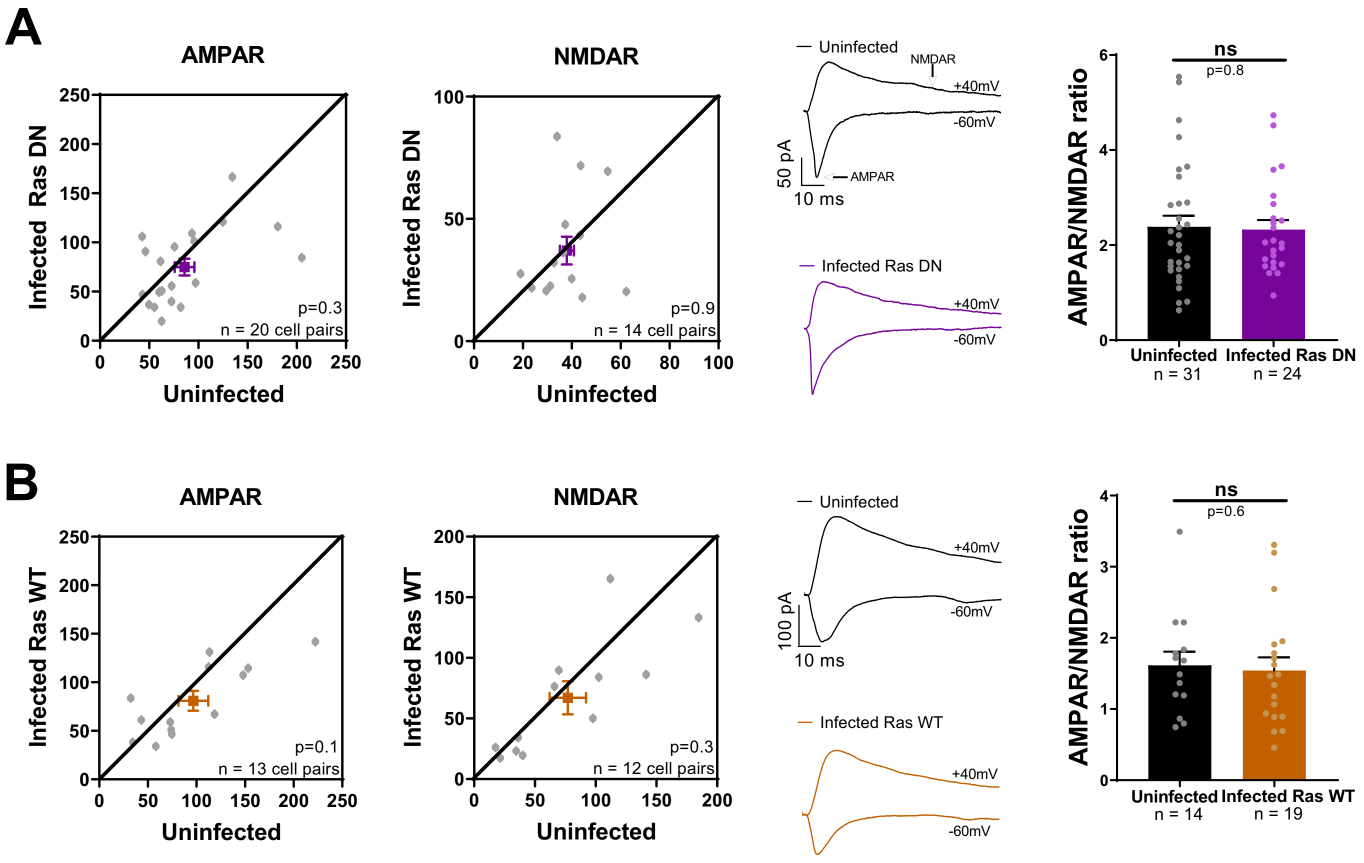

Figure EV1. Effect of Ras dominant negative on basal synaptic transmission.

Scatter plot of simultaneous recordings of AMPAR- or NMDAR-mediated responses from uninfected and Ras-DN (**A**) or Ras WT (**B**) infected neurons (left). Representative traces (middle). Quantification of AMPA/NMDA ratio (right) represented as mean ± SEM; individual values are also plotted. Mann-Whitney test was used to evaluate significant differences. ns, non-significant.

 © The Author(s)

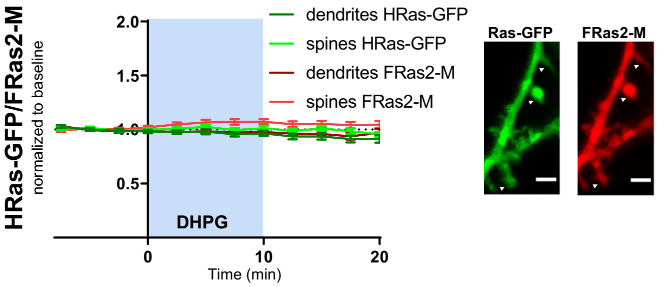

Figure EV2. H-Ras-GFP and FRas2-M fluorescence during DHPG treatment.

Left. Time course of H-Ras-GFP and FRas2-M signal in spines ($n = 50$) and dendrites ($n = 15$) of CA1 pyramidal cells ($n = 10$) upon DHPG stimulation. Results are represented as mean ± SEM. Kruskal–Wallis test was used to evaluate differences across time. [$H(11,571) = 15.1$, $p = 0.2$ for FRas2-M and $H(11,571) = 18.8$, $p = 0.06$ for Ras-GFP]. Right. Representative images of dendritic branches of CA1 neurons coexpressing H-Ras-GFP (green channel) and FRas2-M (red channel) constructs. Scale bars 2 µm. White arrows point dendritic spines.

© The Author(s)

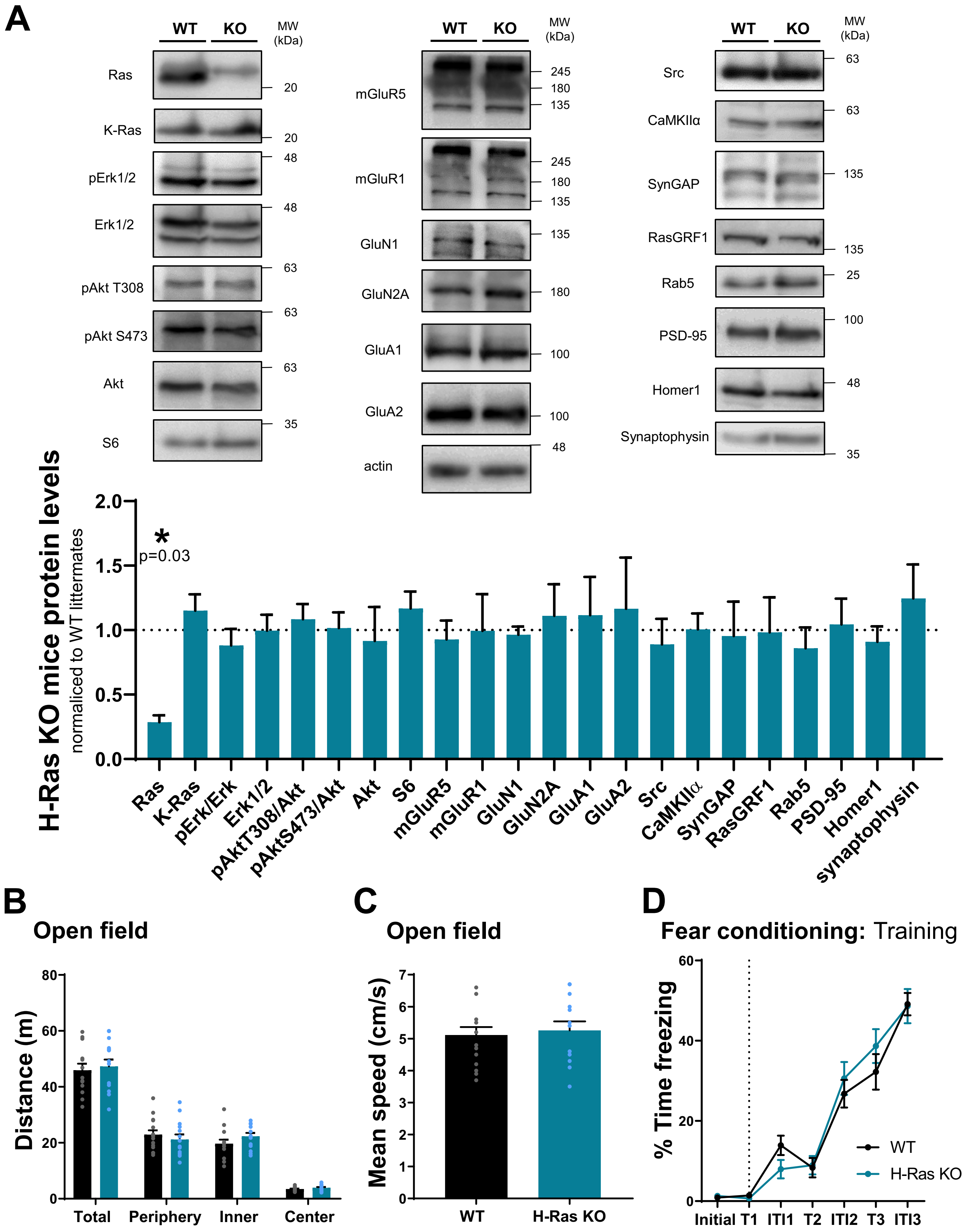
A
WT
KO
MW (kDa)
Ras
K-Ras
pErk1/2
Erk1/2
pAkt T308
pAkt S473
Akt
S6
mGluR5
mGluR1
GluN1
GluN2A
GluA1
GluA2
actin
Src
CaMKIIα
SynGAP
RasGRF1
Rab5
PSD-95
Homer1
Synaptophysin
H-Ras KO mice protein levels
normaliced to WT littermates
p=0.03
Ras
K-Ras
pErk/Erk
Erk1/2
pAktT308/Akt
pAktS473/Akt
Akt
S6
mGluR5
mGluR1
GluN1
GluN2A
GluA1
GluA2
Src
CaMKIIα
SynGAP
RasGRF1
Rab5
PSD-95
Homer1
synaptophysin
B
Open field
Distance (m)
Total
Periphery
Inner
Center
C
Open field
Mean speed (cm/s)
WT
H-Ras KO
D
Fear conditioning: Training
% Time freezing
Initial
T1
ITI1
T2
ITI2
T3
ITI3
WT
H-Ras KO

 © The Author(s)

Figure EV3. Hippocampal levels of different synaptic and signaling proteins and animal behavior in H-Ras KO mice.

(**A**) Representative Western blots (upper) and quantification (lower) of Ras proteins and different Ras effectors, signaling molecules and glutamate receptors. Some blots have been reused from Fig. 5 (inputs of K-Ras, Rab5, PSD-95, synaptophysin), as they come from the same experiment/animal. Unless otherwise indicated, each sample is normalized to actin levels and results are referred to WT animals and represented as mean ± SEM, $n = 6$ animals per condition. Wilcoxon signed-rank test (*) was used to evaluate changes; non-significant differences were found except for total Ras levels. (**B**) Total distance traveled in the open field test and in the different subsections of the arena. Mean ± SEM; individual values are also represented. Mixed effects analysis was used to evaluate differences between genotypes [$F(1,27) = 0.2$, $p = 0.7$]. (**C**) Mean speed in the open field test. Mean ± SEM; individual values are also represented. Mann-Whitney test was used to evaluate differences between genotypes ($p = 0.9$). (**B**, **C**) $n = 15$ mice for WT and 14 mice for H-Ras KO. (**D**) Percentage of time spent freezing during fear conditioning training session. T, tone; ITI, inter-tone interval. Mean ± SEM is represented. Mixed effects analysis was used to evaluate differences between genotypes [$F(1,45) = 0.1$, $p = 0.8$]. $n = 25$ mice for WT and 22 mice for H-Ras KO.

© The Author(s)

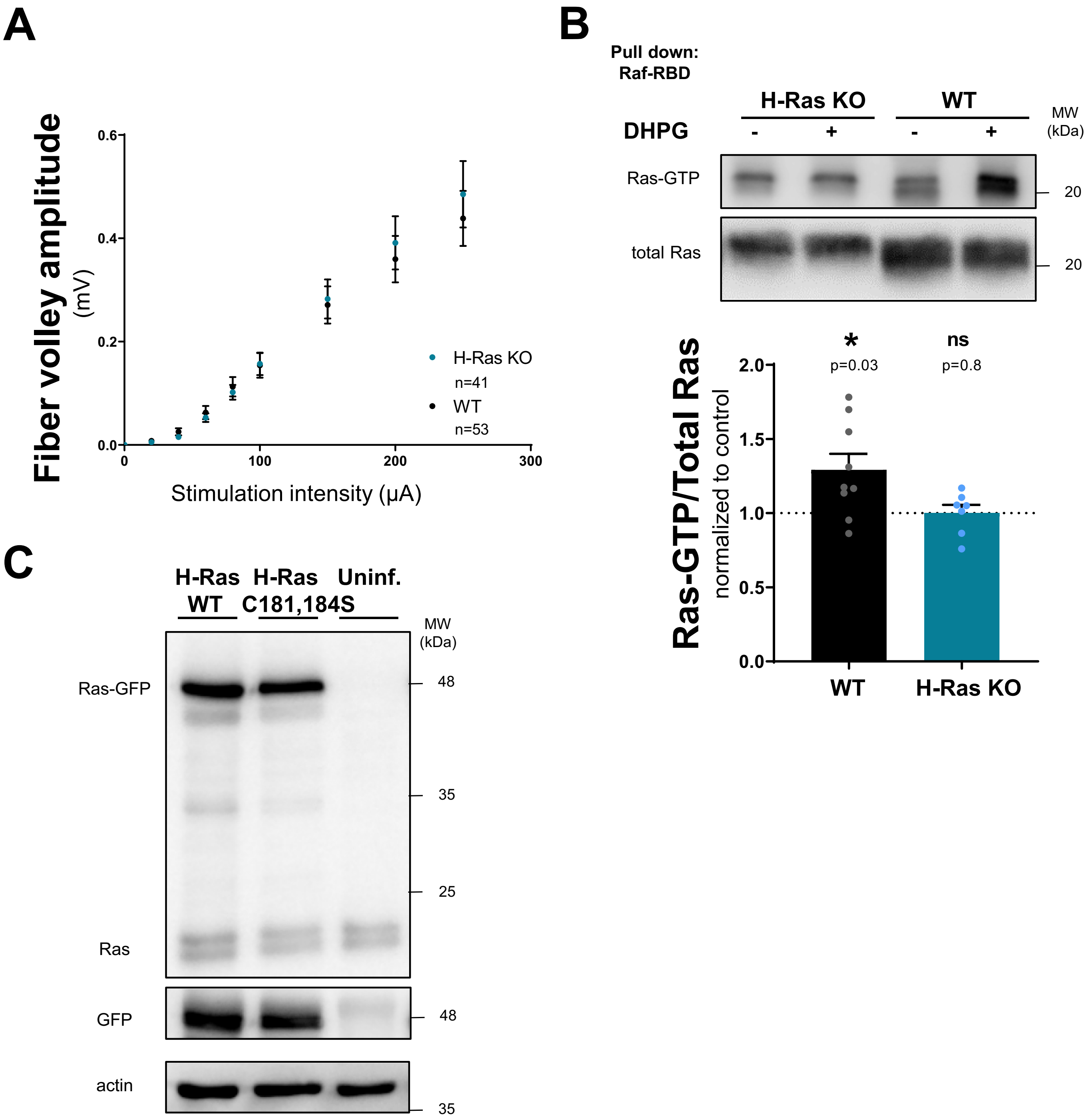

Figure EV4. Analysis of basal transmission and Ras activity in H-Ras KO mice.

(**A**) Fiber volley responses over increasing stimulation intensities during input/output curve measurements shown in Fig. 4A. Mean ± SEM; Mixed effects analysis [F(1,92) = 0.1, $p = 0.8$]. (**B**) Quantification (lower panel) and representative blot (upper panel) of active Ras pulled-down vs total Ras from acute hippocampal slices of WT and H-Ras KO mice 10 min after DHPG treatment. Results are normalized to control non-stimulated slices (in WT or KO mice) and expressed as mean ± SEM, $n = 7$–9 independent slices/mice. Wilcoxon signed-rank test (*) was used to assess statistically the effect of DHPG. ns, non-significant. (**C**) Representative blot of Ras and GFP in organotypic hippocampal slices infected with H-Ras WT and H-Ras C181,184S, or uninfected (uninf.). Note that both endogenous Ras (~21 KDa) and recombinant Ras-GFP (~48 KDa) were detected at their appropriate sizes. Actin was used a protein loading control.

 © The Author(s)

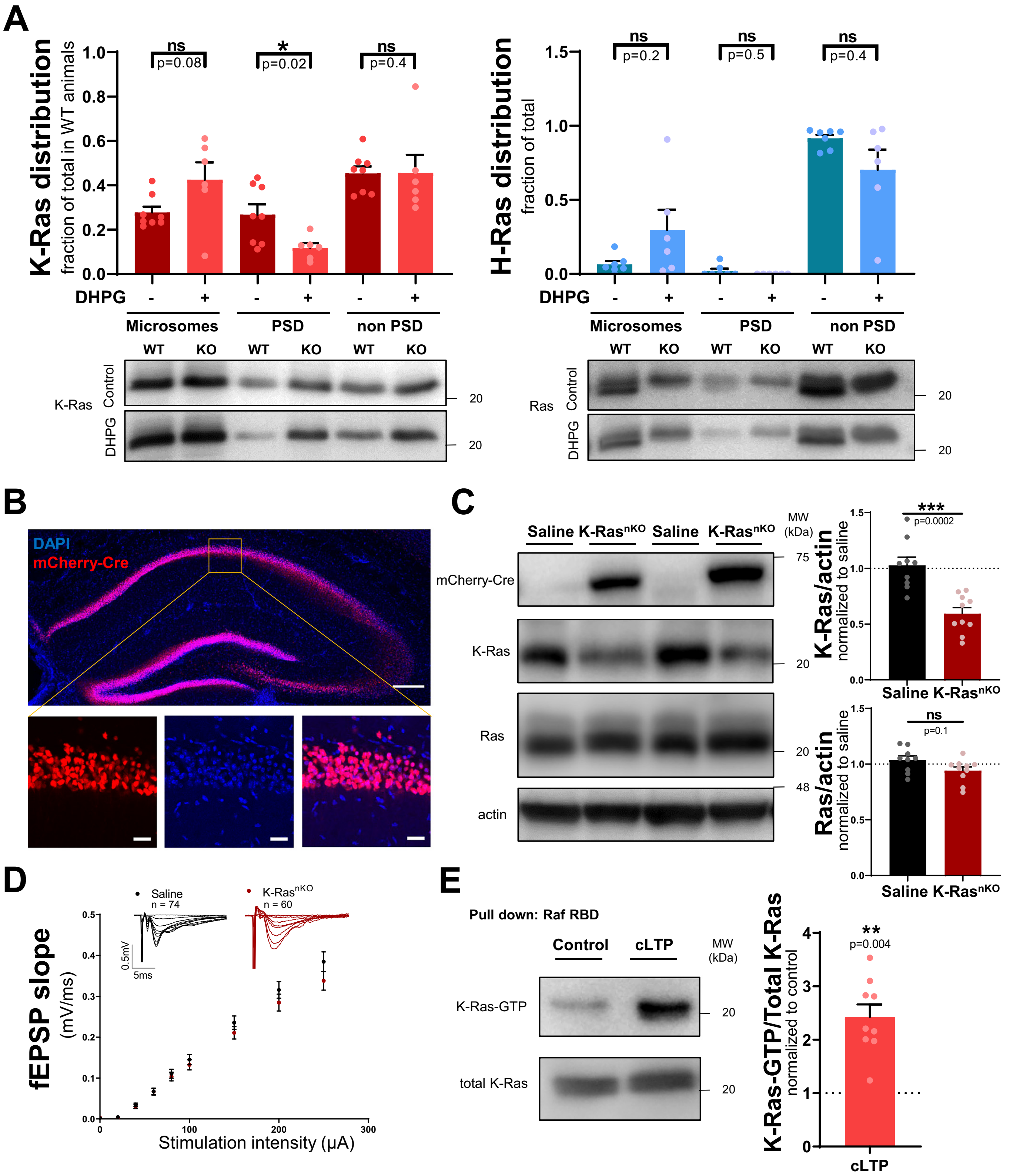
A
K-Ras distribution
fraction of total in WT animals
ns p=0.08
* p=0.02
ns p=0.4
DHPG
Microsomes
PSD
non PSD
WT KO WT KO WT KO
K-Ras
Control
DHPG
20
H-Ras distribution
fraction of total
ns p=0.2
ns p=0.5
ns p=0.4
Ras
B
DAPI
mCherry-Cre
C
Saline K-RasnKO Saline K-RasnKO
MW (kDa)
mCherry-Cre
K-Ras
Ras
actin
75
48
K-Ras/actin normalized to saline
*** p=0.0002
Ras/actin normalized to saline
ns p=0.1
Saline K-RasnKO
D
Saline n = 74
K-RasnKO n = 60
0.5mV
5ms
fEPSP slope (mV/ms)
Stimulation intensity (μA)
E
Pull down: Raf RBD
Control cLTP
K-Ras-GTP
total K-Ras
K-Ras-GTP/Total K-Ras normalized to control
** p=0.004
cLTP

© The Author(s)

Figure EV5. Analysis of Ras distribution, expression, and activity in H-Ras and neuronal K-Ras KO mice.

(**A**) Representative blots (lower pannel) of total Ras and K-Ras in different subcellular compartments following DHPG stimulation in hippocampal slices from WT and H-Ras KO mice. H- and K-Ras distribution in microsomes, PSD and non-PSD fractions was quantified (upper panel) as percentage of total protein (from inputs) for each isoform. H-Ras signal was obtained by subtracting total Ras signal of H-Ras KO mice from that of WT mice. Results expressed as mean ± SEM, $n = 6$–8 animals per condition (controls from Fig. 5A were included in the quantification); individual values are also represented. Mann-Whitney test was used to evaluate DHPG effect in each fraction compared to control, non-stimulated slices. (**B**) Representative image of mCherry fluorescence (red) and DAPI staining (blue) of a hippocampal slice from AAV-CaMKII-mCherry-Cre-infected K-Ras$^{flox/flox}$ mice (lower panels: zoom-in images of CA1 region). Scale bars 200 (upper) and 20 (lower) µm. (**C**) Representative Western blots (left) and quantification (right) of K-Ras and total Ras hippocampal levels. Results are normalized to saline average levels and expressed as mean ± SEM; individual values are also represented. Mann-Whitney test (*) was used to evaluate statistical differences. $n = 9$ (WT) and 10 (K-Ras nKO) mice. (**D**) Input/output curves of fEPSP slopes vs stimulation intensities. Representative traces are shown in the upper part. Mean ± SEM; Mixed effects analysis was used to assess statistical differences between genotypes [$F(1,132) = 1.0$, $p = 0.3$]. (**E**) Quantification (left) and representative blot (right) of active K-Ras pulled-down vs total K-Ras from mouse organotypic hippocampal slices 10 min after cLTP treatment. Results are normalized to control non-stimulated slices and expressed as mean ± SEM, $n = 9$. Wilcoxon signed-rank test (*) was used to assess statistically the effect of cLTP induction. ns, non-significant.

© The Author(s)
